# Tracking the Cracking: A Holistic Analysis of Rapid Ice Shelf Fracture Using Seismology, Geodesy, and Satellite Imagery on the Pine Island Glacier Ice Shelf, West Antarctica

**DOI:** 10.1029/2021GL097604

**Published:** 2022-05-20

**Authors:** S. D. Olinger, B. P. Lipovsky, M. A. Denolle, B. W. Crowell

**Affiliations:** ^1^ Department of Earth and Planetary Sciences Harvard University Cambridge MA USA; ^2^ Department of Earth and Space Sciences University of Washington Seattle WA USA

**Keywords:** ice shelf fracture, Pine Island Glacier, rifting, calving

## Abstract

Ice shelves regulate the stability of marine ice sheets. We track fractures on Pine Island Glacier, a quickly accelerating glacier in West Antarctica that contributes more to sea level rise than any other glacier. Using an on‐ice seismic network deployed from 2012 to 2014, we catalog icequakes that dominantly consist of flexural gravity waves. Icequakes occur near the rift tip and in two distinct areas of the shear margin, and TerraSAR‐X imagery shows significant fracture in each source region. Rift‐tip icequakes increase with ice speed, linking rift fracture to glaciological stresses and/or localized thinning. Using a simple flexural gravity wave model, we deconvolve wave propagation effects to estimate icequake source durations of 19.5–50.0 s and transient loads of 3.8–14.0 kPa corresponding to 4.3–15.9 m of crevasse growth per icequake. These long‐source durations suggest that water flow may limit the rate of crevasse opening.

## Key Points

- Fracture at Pine Island Glacier generates flexural gravity waves, a wave type related to interaction between a floating plate and supporting fluid
- Rift‐tip seismicity rate increases with ice speed either due to changes in the underlying ice shelf stress state or localized thinning
- Recorded flexural gravity waves are consistent with a point load of ∼10 kPa applied over ∼30s, corresponding to ∼10m of vertical cracking

## 1 Introduction

Ice shelf fracture exerts a fundamental control on the stability of marine ice sheets and associated sea level fluctuations (Seroussi et al., 2020). In particular, understanding the past, present, and future stability of the West Antarctic Ice Sheet (WAIS) remains one of the great challenges of modern glaciological research and is itself closely related to ice shelf fracturing processes (Scambos et al., 2017). Fractures on ice shelves take on many forms, including through‐cutting rifts (Hulbe et al., 2010; Larour et al., 2004; Lipovsky, 2020), smaller‐scale basal and surface crevasses (McGrath et al., 2012; Rist et al., 2002), hydraulic fracturing (Banwell et al., 2013; Weertman, 1973), and cliff failure (Clerc et al., 2019). Despite decades of progress, understanding of ice shelf fracture remains significantly hindered by a lack of direct observation (Benn et al., 2007). A number of basic questions remain or have only partially been addressed: What forces are involved in ice shelf fracture? Is ice shelf fracture a fast and brittle or slow and ductile process? To what degree is water involved in fracture propagation? Does ice shelf fracture growth happen at a constant rate or in bursts, and what controls its timing?

All of these questions can be addressed using seismology. Because seismic waves carry information about the dynamics of fracture, numerous previous studies have leveraged such signals, referred to as icequakes, for this purpose (Chen et al., 2019; Hammer et al., 2015; Von der Osten‐Woldenburg, 1990; Winberry et al., 2020). Seismic studies on ice shelves have shown that crevasse propagation is intermittent (Bassis et al., 2007; Heeszel et al., 2014) and has highlighted environmental forcing that would be difficult to ascertain using only remotely sensed observations (Aster et al., 2021; Bassis et al., 2008; Olinger et al., 2019).

Here, we use seismic recordings to quantify fracturing of Pine Island Glacier (PIG) Ice Shelf. PIG, part of the larger WAIS, contributes more to present‐day global sea level rise than any other glacier (Shepherd et al., 2018). Current ice mass loss at PIG is thought to be due to the retreat of the floating ice shelf (Joughin, Shapero, Smith, et al., 2021) being caused by interactions between ocean forcing (Christianson et al., 2016; Joughin, Shapero, Dutrieux, & Smith, 2021) and fracturing processes (MacGregor et al., 2012).

We focus on icequakes that travel as flexural gravity waves to quantify the fracturing of PIG Ice Shelf. Flexural gravity waves are a type of hybrid seismic‐water wave (Ewing & Crary, 1934) unique to floating structures, such as ice shelves, since both elasticity and buoyancy act as their restoring force (Ewing & Crary, 1934). Flexural gravity waves are strongly dispersive (Ewing & Crary, 1934), which can make waveform analysis difficult and necessitate careful modeling (Lipovsky, 2018; Mattsson et al., 2018; Sergienko, 2017). Despite this challenge, flexural gravity waves are useful tools to study ice shelf processes because while direct body waves in ice shelves are often not observed at distances greater than a few ice thickness (Zhan et al., 2014), flexural gravity waves are often observed to travel long distances from their exciting source (Williams & Robinson, 1981).

Many sources generate flexural gravity waves on ice shelves, including ocean swell (Williams & Robinson, 1981), tsunamis (Bromirski et al., 2017), and airplane landings (MacAyeal et al., 2009). MacAyeal et al. (2009) appear to have been the first to propose that fracturing processes in ice shelves may act as seismic sources that generate flexural gravity waves. MacAyeal et al. (2009) considered water motion in a deforming rift and motion of detaching blocks from the ice front as two such sources. Here, we hypothesize that crevasse growth generates flexural gravity waves.

We begin our fracture analysis by describing a timeline of events with the use of satellite imagery. Next, we catalog flexural gravity waves on PIG to examine the relationship between crack growth, large‐scale rift propagation, shear margin processes, and ice shelf acceleration. We then interrogate icequake source physics by modeling the ice shelf as a buoyantly supported beam, the simplest model that captures flexural gravity wave propagation (Mattsson et al., 2018; Sergienko, 2017). In our analysis, we model flexural gravity wave generation by a point load or bending moment applied during ice shelf crevasse growth to infer key source parameters of the recorded icequakes.

## 2 Analysis of Satellite Imagery and Positioning

**Figure 1:**
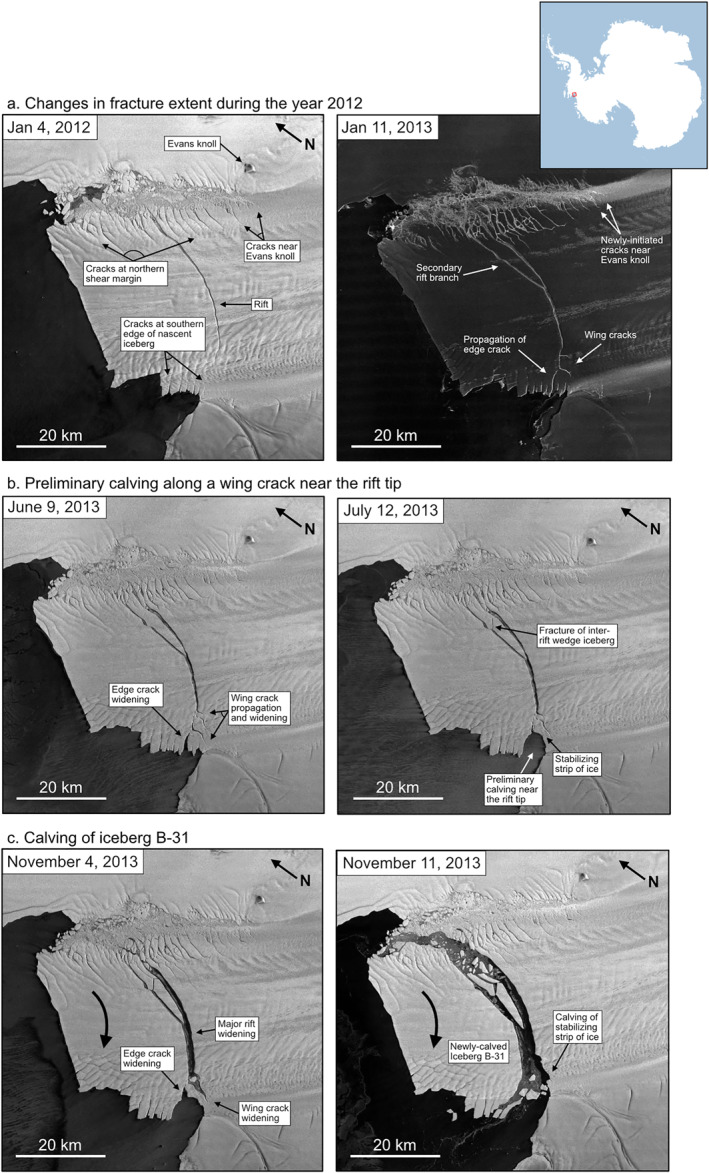
TerraSAR‐X images showing an overview of fracture development at Pine Island Glacier (PIG) from 2012 to 2014. Large arrows in panel c show sense of motion of the iceberg. See text for full discussion. Inset shows the location of PIG in Antarctica.

We track visible fracturing on PIG using images collected by the TerraSAR‐X satellite (Pitz & Miller, 2010) from 2012 to 2014. At the start of our study period in January 2012, the primary visible fractures are the rift, ∼20 large cracks extending into the ice shelf from northern shear margin, and ∼10 cracks extending into the ice shelf at the southern edge of the nascent iceberg (Figure 1a, left). By January 2013, the rift had propagated a few kilometers without significant widening, and two wing cracks (Renshaw & Schulson, 2001) opened at the rift tip (Figure 1a, right). One of the cracks at the northern shear margin extended 7 km and connected to the rift between 8 May and 11 May 2012. The other northern shear margin cracks extended and widened, at least two new cracks initiated near Evans Knoll, and one of the cracks at the southern edge of the nascent iceberg extended to within a kilometer of the rift tip.

During the first four months of 2013, the wing cracks near the rift tip extended and widened. In early July 2013, a block of ice calved along a wing crack at the southern edge of the nascent iceberg near the rift tip (Figure 1b). After this preliminary calving event, the only connection between the nascent iceberg and the ice shelf was a 2‐km‐wide strip of ice between the ocean and a wing crack. Over the next few months, we observe significant widening of the rift likely due to the iceberg beginning to drift away from the ice shelf. Iceberg B‐31 calved in November 2013 (Figure 1c) when left lateral motion of the iceberg pried opens a large wing crack near the rift tip until a strip of ice stabilizing the iceberg broke off, allowing Iceberg B‐31 to drift into the sea. By the end of 2013, many fractures in the northern shear margin had extended and calved smaller icebergs, and several new fractures had initiated near Evans Knoll.

**Figure 2:**
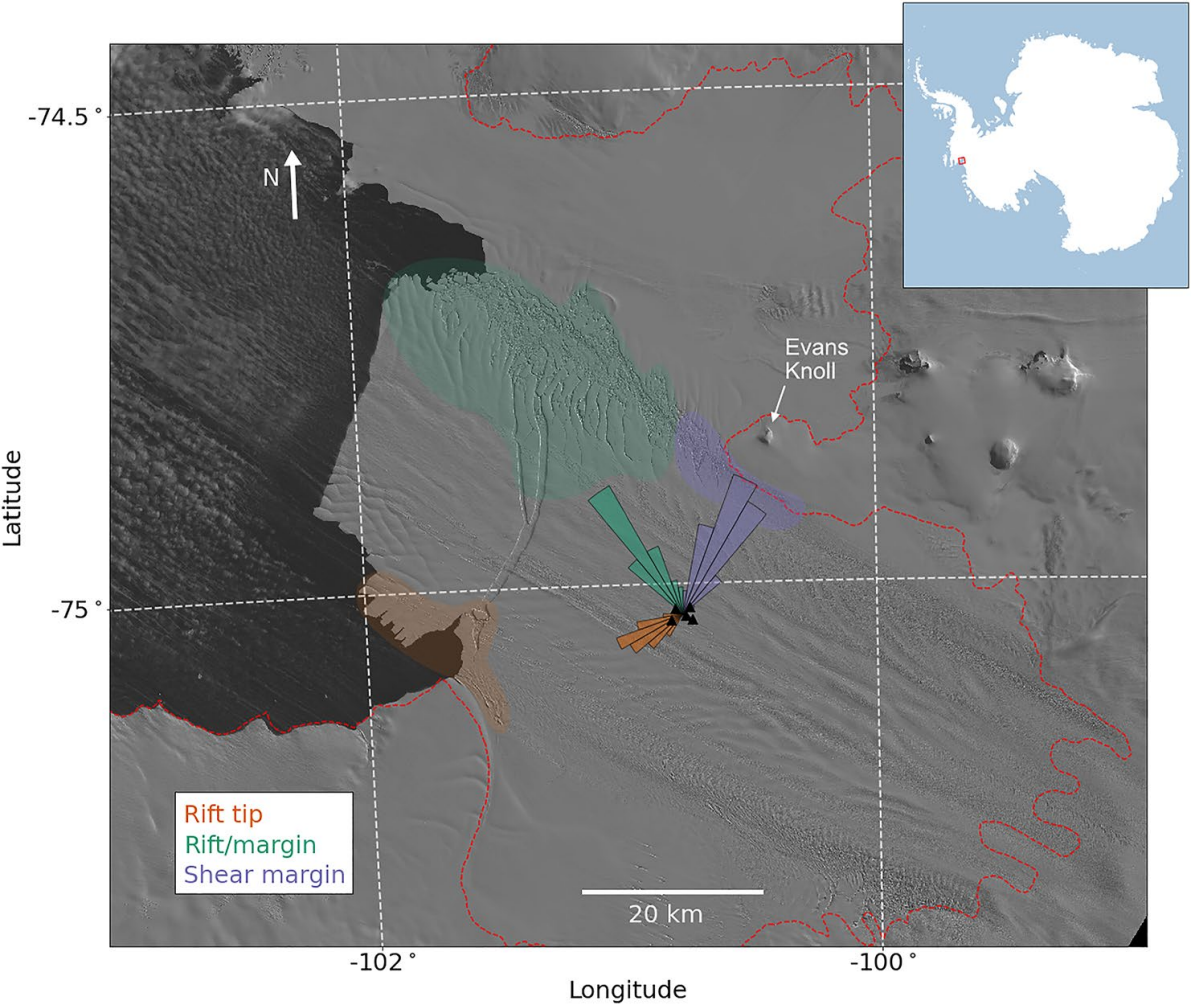
Back‐azimuthal histogram showing locations of cataloged icequake. Rift‐tip event back azimuths are plotted as orange rays. Rift/margin event back azimuths are plotted as green rays. Shear‐margin event back azimuths are plotted as purple rays. Likely source regions are shown by colored polygons. Pine Island Glacier array seismic and Global Positioning System stations are plotted as black triangles. Approximate grounding line position is shown by the red‐dashed line (Bindschadler et al., 2011). Background LANDSAT imagery is from October 2013 (courtesy of the United States Geological Survey).

**Figure 3:**
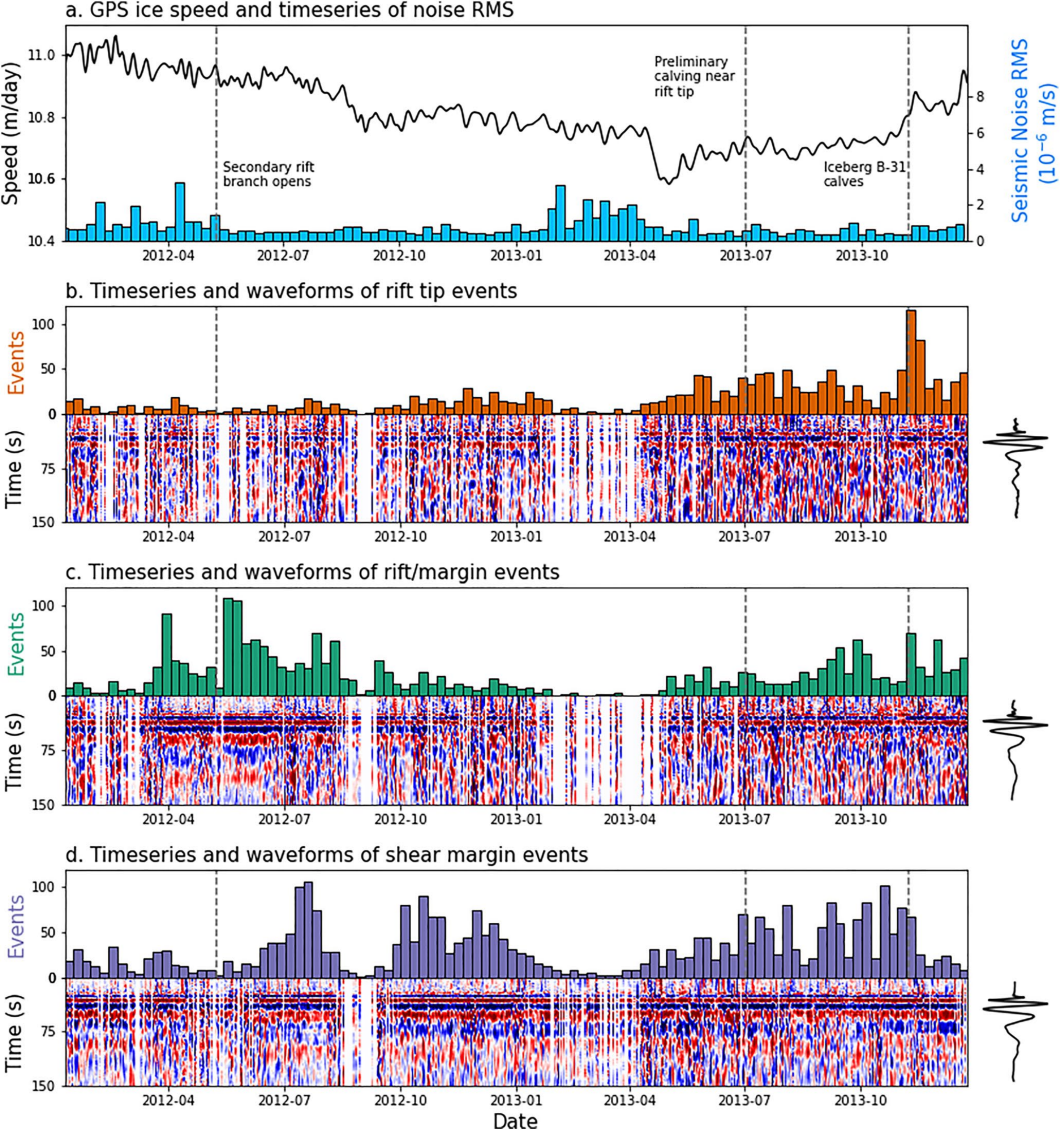
Timing and waveforms of cataloged icequake. (a) Global Positioning System (GPS)‐derived ice velocity is shown by the black line, and the average seismic noise is shown by blue bars. Noise is highest in the Antarctic Summer, when minimal sea ice is present to attenuate ocean‐generated noise, reducing detectability in January, February, and March. (b) Rift‐tip events. Weekly time series of rift‐tip event times is shown by orange bars. Daily vertical (HHZ) waveform stacks of detected rift‐tip events are plotted beneath. Overall rift‐tip event stack is shown to the right. (c) Same as (b) for rift/margin events, color‐coded in green. (d) Same as (b) for shear‐margin events, color‐coded in purple.

We examine Global Positioning System (GPS) speed time series derived from five continuous GPS stations. The GPS stations were colocated with seismometers (locations shown in Figure 2). Our GPS processing is described in Text S1 in Supporting Information S1. Figure 3a plots the GPS‐derived ice shelf speed. We find that ice speed at PIG decreases from 11.1 m/day in January 2012 to 10.8 m/day in April 2013. Then, ice speed drops to below 10.6 m/day for eight days in early May 2013. Following this rapid slowdown, ice speed begins to increase, reaching 10.9 m/day by the end of 2013. The GPS ice speed we compute here is consistent with a previous study utilizing the same data set (Christianson et al., 2016).

## 3 Analysis of Seismograms

We examine seismic data from five sites on PIG (Stanton et al., 2013). The instruments were deployed in January 2012 and retrieved in December 2013, providing two years of continuous data. The seismic stations were deployed in a cross shape with 5 km aperture at the center of the ice shelf (Figure 2). Each site consisted of a three‐component Nanometrics Trillium 120 Broadband seismometer and a Quanterra Q330 digitizer (Holland & Bindschadler, 2012). Seismic data were sampled at 100 Hz, and we removed the instrumental response on the frequency band 0.001–45 Hz.

In the seismic data set, we observe events with an abrupt onset and with high frequencies that arrive before low frequencies. This type of dispersion is a characteristic of flexural gravity waves. The observed dispersion (high‐frequency waves travel faster) is the opposite of typical surface waves in the solid Earth. In the latter case, low‐frequency waves travel faster because seismic wave speeds generally increase with depth.

To detect flexural gravity waves in the data set, we design a two‐stage detection scheme that identifies broadband, dispersive seismic events. Our detection approach, detailed in Text S2 in Supporting Information S1, uses a dual‐band short‐term average/long‐term average (STA/LTA) detector in combination with template matching (Allen, 1978; Gibbons & Ringdal, 2006) to detect a preliminary catalog of 22,119 events. Inspection of the preliminary catalog reveals two main families of events: One with clear high‐frequency‐first dispersion and other that is dominantly monochromatic. To isolate flexural gravity waves, we undertake waveform clustering using a K‐Shape algorithm (Paparrizos & Gravano, 2016) modified to operate on multicomponent seismic data. Visual analysis of the clustered catalog demonstrates the efficacy of our approach in isolating flexural gravity waves (Figure 3). Our final catalog contains 8,184 likely flexural gravity wave events, which we refer to as icequakes in the rest of the text.

We next determine locations for all icequakes in our final catalog. Given the poor distribution of the stations with respect to fracture locations, we employ single‐station approaches to locating icequakes. We compute epicentral back azimuths by analyzing the polarization direction of recorded horizontal waves (Aster et al., 2021). We apply principle component analysis (PCA) to the horizontal‐component seismograms to retrieve polarization directions. The polarization provides a 180° ambiguity, so we find the direction of propagation based on which station recorded the first arrival (see Text S3 in Supporting Information S1).

We locate all of the 8,184 icequakes to one of three distinct source regions: the rift tip, the body of the rift and nearby shear margin (“rift/margin”), and the northeast shear margin near Evan's knoll (“shear margin”), which are depicted in Figure 2. These spatial groups correspond to 22%, 29%, and 40% of the catalog, respectively, with 9% of events having indeterminate locations. Figure 2 shows the back‐azimuthal histograms of the three groups.

## 4 Relationships Between Icequakes and Ice Shelf Behavior

### 4.1 Rift Tip

The rift‐tip icequakes are coincident in space and time with several fracturing processes, including rift propagation, wing cracking, small‐scale calving within the rift, and calving along the southern edge of the nascent iceberg. Rift‐tip events occurred more frequently in 2013 than in 2012 (Figure 3b). The mean seismicity rate was 9.4 icequakes/week in 2012 and 25.6 icequakes/week in 2013. 19 weeks of 2013 equaled or exceeded the maximum 2012 seismicity rate of 29 icequakes/week. Weekly icequake counts increased past the peak level seen in 2012 on 21 May 2013 and remain elevated until the end of the deployment. This period of elevated rift‐tip seismicity corresponds to the phase of significant wing crack growth and rift widening observed in imagery. Rift‐tip icequakes appear located ∼15° south of the rift tip's position in LANDSAT imagery from 12 October 2013 (Figure 2). However, when Iceberg B‐31 calved in November 2013, the wing crack extending south of the rift had propagated to a location consistent with the peak in the back‐azimuthal distribution of rift‐tip icequakes (Figure 1c, left).

Peak levels of rift‐tip seismicity were observed during the calving of Iceberg B‐31 in the week of 5 November 2013. That week had 115 rift‐tip events, the highest event count of any week across all three source regions. Furthermore, elevated rift‐tip icequake activity in 2013 corresponds to a period of accelerating ice velocities (Figure 3a). While rift‐tip fracture may be more directly related to strain rate in a viscous regime and strain in an elastic regime, we simply note that ice speed reflects the underlying stress state of the ice shelf. The correspondence in time between elevated rift‐tip seismicity rates and increasing ice velocities therefore suggests that rift propagation is sensitive to the underlying stress state of the ice shelf. In addition, rift‐tip fracture may be enhanced by localized ice shelf thinning and melt within the rift. Christianson et al. (2016) hypothesize that the overall pattern of ice velocities at PIG in 2013 tracks a time‐lagged response to ocean melting, and localized melt has been proposed as a primary driver of rifting at PIG (Jeong et al., 2016; Walker & Gardner, 2019). The observed connection in time between rift‐tip fracture and accelerated ice velocities demonstrates that rift growth and PIG are sensitive to changes in ice dynamics, localized melt, or a combination of both. At the present time, however, we are unable to confirm whether local or more distant melt‐related feedbacks are responsible for the observed fracturing.

### 4.2 Rift/Margin

The rift/margin icequakes are coincident in space and time with the growth of ∼20 rifts formed in the northwest shear zone as well as smaller‐scale fractures and widening of the main rift itself. Rift/margin icequakes occurred more frequently in 2012 than in 2013. The mean seismicity rate was 27.7 icequakes/week in 2012 and 19.3 icequakes/week in 2013. Four weeks of 2012 equaled or exceeded the maximum 2013 seismicity rate of 70 icequakes/week. The timing of icequakes in the rift/margin group is independent of ice speed. Peak levels of rift/margin seismicity were observed during the week of 15 May 2012, which contained 109 rift/margin icequakes. Rift/margin icequakes reach peak seismicity rates in the weeks following the opening of the secondary rift branch in May 2012, suggesting that the crack opening caused aftershock‐like seismicity and/or destabilized the margin, enhancing the growth of nearby fractures.

### 4.3 Shear Margin

The shear‐margin icequakes are coincident in space and time with the initiation of new cracks and growth of extant cracks near Evans Knoll. This area marks the transition from a primarily intact shear margin upstream of Evans Knoll to a highly fractured shear margin downstream of Evans Knoll. Imagery shows that multiple fractures longer than 1 km were initiated in this area during 2012 and 2013 (Figure 1). Shear‐margin icequakes occurred at an approximately equal rate in 2012 and 2013. The mean seismicity rate was 31.9 icequakes/week in 2012 and 32.2 icequakes/week in 2013. Peak levels of shear margin seismicity were observed during the week of 17 July 2012, which contained 107 shear‐margin icequakes. Shear‐margin icequakes do not exhibit any prominent temporal trends and appear independent of ice velocity. The shear margin experiences the highest overall level of seismic activity, suggesting that the transition point from intact to fractured ice near Evans Knoll experiences higher stress concentrations than either the rift tip or the rift/margin regions, consistent with rift modeling (Lipovsky, 2020).

## 5 Icequake Source Analysis

We next estimate the distribution of forces that gives rise to the observed seismograms. We do this by removing wave propagation effects from the observed seismograms using a numerically computed Green's function. Our catalog was designed to represent icequakes that mostly consist of flexural gravity waves. We therefore model the vertical seismograms using the simplest model that gives rise to flexural gravity waves and the dynamic floating beam equation (Ewing & Crary, 1934; Squire & Allan, 1977),

(1) $$\rho_{i}h_{i}\frac{\partial^{2}w}{\partial t^{2}}+D\frac{\partial^{4}w}{\partial x^{4}}+\rho_{w}gw+\rho_{w}\frac{\partial \phi }{\partial t}=P,$$

where $D\equiv EI=Eh_{i}^{3}/\left[12\left(1\nu^{2}\right)\right]$ is the flexural rigidity with the second moment of area $I=\int_{{-h}_{i}/2}^{h_{i}/2}z^{2}dz$, *E* is the Young's modulus of ice, *ν* is the Poisson's ratio of ice, *t* is time, *x* is horizontal position, *g* is gravitational acceleration constant, *h*_*i*_ is the ice thickness, *ρ*_*i*_ is the density of ice, *ρ*_*w*_ is the density of water, *w* is the vertical displacement of the beam, *ϕ* is the ocean surface velocity potential, and *P* is an applied point load. From left to right, the terms in Equation 1 represent inertia, flexure of the ice shelf, buoyancy, and ocean surface waves generated at the ice‐water interface. We initially use a locally averaged ice thickness of *h*_*i*_ = 400 m (Shean et al., 2019) and a water depth of *h*_*w*_ = 590 m (Fretwell et al., 2013).

We model icequake sources as either an applied point load or point bending moment. When a basal crevasse opens and fills with water, the downward‐acting ice overburden stress at the top of the crevasse is greater in magnitude than the upward‐acting buoyancy stress exerted by water filling the crevasse. This applies a downward point load to the ice shelf. In addition, the horizontal ice overburden stress along the walls of the crevasse is greater in magnitude than the horizontal buoyancy stress exerted by the water filling the crevasse. The difference in magnitude between these two stresses decreases with depth such that the walls of a crevasse are subject to stress gradient. This applies a bending moment to the ice shelf. These two mechanisms may act in concert and simultaneously apply a moment and point load to the ice shelf. We choose not to pursue such hybrid sources at the present time because the simplicity of our model—specifically the assumptions of uniform ice thickness and two‐dimensional geometry—suggests that additional source complexity is not warranted prior to improvements in these other areas.

We obtain the Green's function of the floating beam equation as the impulse response of the mechanical system to a point load (force per unit length) source. Rewriting Equation 1 using the linear operator A as Aw=P, the Green's function equation can then be written as AG=δ(x)δ(t). In Text S4 in Supporting Information S1, we derive a frequency‐wavenumber solution for *G* that we are able to analytically invert in the time domain and numerically invert in the frequency domain. We then derive *G*_*m*_, the vertical displacement response to a point moment source.

**Figure 4:**
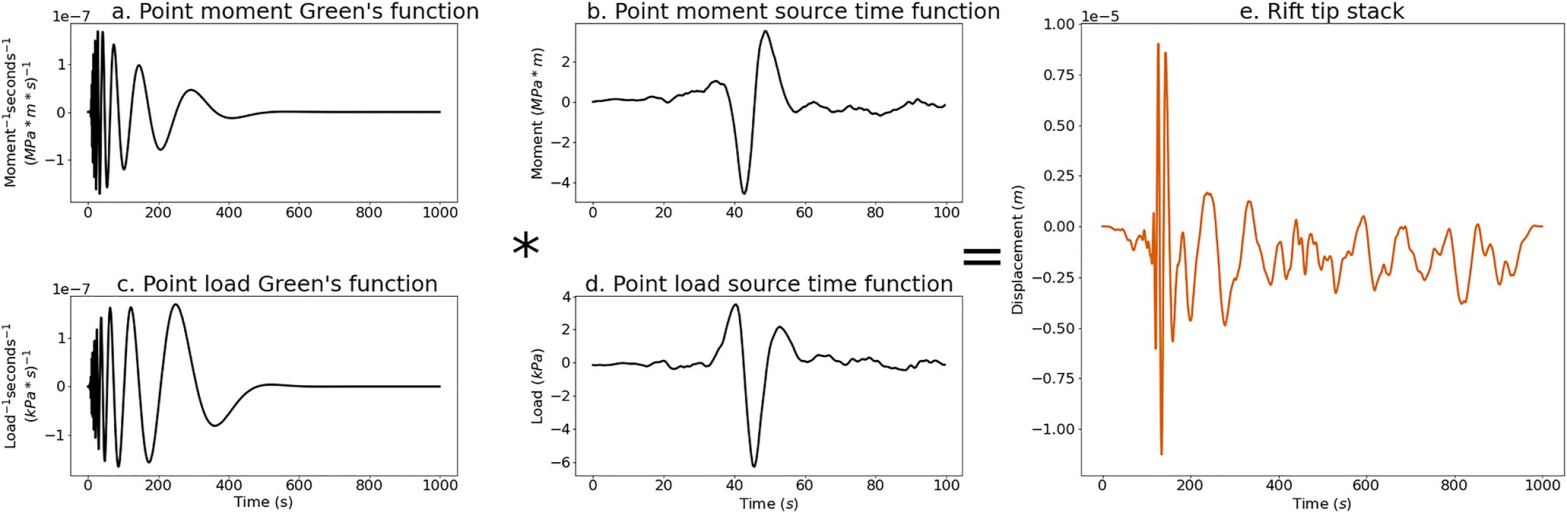
Green's functions and source time functions for rift tip events. (a) Theoretical Green's function for a point moment source located at a distance of 25 km, which is approximately the distance from Pine Island Glacier seismic array to the rift tip. (b) Source time function retrieved by deconvolving the point moment Green's function from the stack of rift tip vertical displacement waveforms. (c) Theoretical Green's function for a point load source located at a distance of 25 km. (d) Source time function retrieved by deconvolving the point load Green's function from the stack of rift tip vertical displacement waveforms. (e) Stack of rift tip vertical displacement waveforms obtained by aligning waveforms to a master event and taking the mean waveform on the frequency band 0.01–1 Hz.

We deconvolve *G* and *G*_*m*_ from waveform stacks to estimate the source load or moment distribution of events in each spatial group. Figure 4 shows our deconvolution result for the rift‐tip icequakes, illustrating that a given vertical displacement seismogram may equivalently be represented as a point moment (Figures 4a and 4b) or a point load (Figures 4c and 4d). The equivalent analysis for the other two groups of events is given in Figures S1 and S2 in Supporting Information S1.

We examine the sensitivity of our deconvolution to the assumed value for the ice thickness by varying the ice thickness between 300 and 500 m (Figures S3–S5 in Supporting Information S1). For the rift‐tip group, we find source durations ranging from 30.48 to 50.00 s and amplitudes ranging from 2.69 to 6.90 MPa⋅m (point moment) and 3.83–8.62 kPa (point load). For the rift/margin group, we find source durations ranging from 19.52 to 48.57 s and amplitudes ranging from 3.82 to 12.55 MPa⋅m (point moment) and from 5.05 to 14.02 kPa (point load). Finally, for the shear‐margin group, we find source durations ranging from 27.14 to 36.67 s and amplitudes ranging from 5.60 to 14.89 MPa⋅m (point moment) and from 8.04 to 12.97 kPa (point load).

## 6 Discussion of Icequake Source Physics

How large were the cracks that generated the recorded flexural gravity waves? We estimate the amount of vertical crack opening for each spatial group using the point load source amplitudes (Text S5 in Supporting Information S1) for ice thickness varying between 300 and 500 m. Rift‐tip point load amplitudes correspond to 4.3–9.8 m of vertical crevasse opening. Rift/margin point load amplitudes correspond to 5.7–15.9 m of vertical crevasse opening. Shear‐margin point load amplitudes correspond to 9.1–14.7 m of vertical crevasse opening. This suggests that the large‐scale fracture opening and rift propagation observed in imagery (Figure 1) were the result of many discrete crack opening events that each spanned only about 1% of the ice thickness, not the result of full‐thickness crack opening. Bassis et al. (2007) and Heeszel et al. (2014) observed episodic rift seismicity on the Amery Ice Shelf and proposed that rifts might propagate due to the coalescence of smaller cracks. Our findings support the hypothesis that crack coalescence can act as a mechanism of rifting.

Estimated source time series for moment and point load exhibit one or several pulses of activity followed by a return to zero (Figure 4). Source time functions derived from body waves in an elastic medium result in estimates of moment rate (Aki & Richards, 2002, Equation 4. 32). Here, however, our deconvolution is sensitive not to the rate of change of point load or moment, but instead to a point load and moment. This complicates the interpretation of the estimated source time series because it suggests that the icequakes represent the application and subsequent removal of some point load or moment. This physically counterintuitive situation motivates an examination of the sensitivity of our deconvolution to static offsets. We therefore calculate synthetic seismograms forced by a step in moment or point load (Figures S6–S8 in Supporting Information S1). We find that in some cases, the step function provides an acceptable fit to the observations. We therefore are unable to infer whether the observed flexural gravity waves were generated by a pulse‐like or step‐like source.

The timescale of the source process, however, is constrained independent of the exact force distribution assumed in the deconvolution. Our source analysis implies that the recorded flexural gravity waves were generated by fracturing process with approximately 20–50 s duration. At this timescale, the observed waves must have been generated by brittle fracture, not by viscous deformation. This 20–50 s timescale is extremely slow compared, for example, to tectonic earthquakes, where earthquake duration scales like 10^*M*/2^ with earthquake moment *M* and 20 s duration are associated with an *M* = 7 earthquake (Ekström et al., 2003).

What process sets the duration of the observed icequakes? The above scaling for tectonic earthquakes is based on the reasoning that the duration is set by the time required for a shear crack to propagate across a fault of length *L* at a rate that tends toward inertial velocities (either the shear or dilatational wave speed *v*_*s*_ or *v*_*p*_) (Freund, 1998). In our system, however, we expect that water plays a limiting role in the speed of fracture propagation that may not be present in tectonic earthquakes. The propagation of fluid filled basal crevasses is expected to occur at the crack wave speed (Lipovsky & Dunham, 2015). The crack wave speed is much slower than the inertial velocities and could plausibly be in the range of 1–100 m/s for basal crevasses in ice shelves. These velocities would suggest source length scales on the order of meters to hundreds of meters. A second plausible explanation is that long durations may be explained by the coalescence of many smaller individual fractures that open successively. And yet another explanation is that there could be significant horizontal propagation, which is not captured in our model. We expect that more detailed near‐source observations would be able to distinguish between these possible scenarios.

## 7 Conclusions

We detect and locate icequakes that propagate as flexural gravity waves on the Pine Island Glacier ice shelf from 2012 to 2014. When compared to satellite imagery, the back‐azimuthal distribution of the detected events suggests that the icequakes were generated by fractures at the tip of a large rift and in two distinct portions of the northern shear margin. Most of the events were generated at the shear margin near Evans Knoll in agreement with imagery that suggests significant fracture initiation. Increased fracturing at the rift tip is associated with increased ice speed and elevated basal melting in 2013 (Christianson et al., 2016). We attribute this relationship to changes in the stress state of the ice shelf or to melt‐driven thinning that elevated rift‐tip stress concentrations. We use a simple model of flexural gravity waves to constrain the source of the recorded waves. We find that the observed waves have a source duration between 20 and 50 s. This timescale implies that a brittle fracture process generated the waves. Our analysis therefore confirms the role of brittle processes in the long‐term evolution of marine ice sheets.

## Supporting information

Supporting Information S1

## Data Availability

Data collected are available through the IRIS Data Management Center. TerraSAR‐X images were obtained using the freely available EOWEB GeoPortal courtesy of the German Aerospace Center (DLR). Code to reproduce the processing workflow for this paper is hosted at https://zenodo.org/badge/latestdoi/468875023.
