# Supplementary material for: Tracking the Cracking: A Holistic Analysis of Rapid Ice Shelf Fracture Using Seismology, Geodesy, and Satellite Imagery on the Pine Island Glacier Ice Shelf, West Antarctica
Source: Geophys Res Lett. 2022 May 20;49(10):e2021GL097604. doi: 10.1029/2021GL097604 (PMC9285369; doi:10.1029/2021GL097604)
Supplement: Supplementary file 1 — Supporting Information S1 [file GRL-49-0-s001.pdf]

Supporting Information for "Tracking the Cracking: a Holistic Analysis of Rapid Ice Shelf Fracture Using Seismology, Geodesy, and Satellite Imagery on the Pine Island Glacier Ice Shelf, West Antarctica"

S. D. Olinger[1,2], B. Lipovsky[2], M. Denolle[2], B. Crowell[2]

[1]Department of Earth and Planetary Sciences, Harvard University, Cambridge, Massachusetts, USA

[2]Department of Earth and Space Sciences, University of Washington, Seattle, Washington, USA

Contents of this file

1. Text S1 to S5
2. Figures S1 to S8
3. Table S1

1. Text S1, GPS Processing

We processed five continuous GPS stations in the region, BOAR and SOW1-4 from 2012 to 2014. Each station was positioned kinematically in the International Terrestrial Reference Frame (ITRF) at a 30 s sample rate with GipsyX, using final Jet Propulsion Laboratory orbits. Ocean tidal loading and solid Earth tides were not removed from the derived displacement time series as these terms are needed to obtain the full glacial

dynamics. After obtaining the 30 s ITRF solutions, we performed a 5 min weighted average using the inverse of the individual epoch uncertainties for data weights, and then rotated the XYZ displacements into local North, East, and up displacements.

We obtain ice speed from the processed GPS positions at the GPS station SOW3 by calculating the total distance moved in each day of the deployment and differentiating with respect to time. The resulting ice speed curve contains some spike artifacts that arise from numerical differentiation, which we remove by linearly interpolating between the ice speed before and after the affected time period. Finally, we low pass filter the data to remove trends on time periods shorter than a week.

2. Text S2, Seismogram analysis

2.1. Icequake detection

To detect flexural gravity icequakes in the dataset, we design a two-stage detection scheme that identifies broadband, dispersive seismic events. First, we employ a short term average/long term average (STA/LTA) impulsivity detector. This method identifies high-amplitude impulsive events by comparing the mean amplitude of a short time window with the mean amplitude of a long time window (Allen, 1978). The detector is triggered when STA exceeds LTA by some threshold. STA/LTA threshold values are selected by tuning the algorithm to successfully detect high signal-to-noise ratio manually-identified events (see Table S1). We carry out STA/LTA on the vertical component of each station separately in two different frequency bands (0.01-1 Hz and 1-10 Hz). Selected waveforms satisfy the STA/LTA trigger criteria in both frequency bands on at least three out of the five stations. We refine the catalog and generate waveform templates by cross-correlating

each preliminary event with a master event waveform and selecting the events with cross correlation coefficients exceeding 0.9. This selection procedure resulted in 57 template events.

Second, we perform a template matching technique based on cross-correlation to identify events that were similar to the events in the preliminary catalog (Gibbons & Ringdal, 2006). To detect new events, each template event is cross correlated with all time windows in the dataset on two frequency bands (0.05-1 Hz and 1-10 Hz). We increase the lower frequency bound from 0.01 Hz to 0.05 Hz since many template events contained uninterpretable noise at frequencies below 0.05 Hz. The detector is triggered when the cross-correlation coefficient between a template event waveform and the given time window exceeds a threshold. The threshold value is selected so that the algorithm successfully detects the other known events of the preliminary catalog (see Supporting Table S1). Detected waveforms satisfy the trigger criteria on at least three out of the five stations in both frequency bands. We carry out this procedure for each template and removed redundant detections to yield the final catalog.

We detect 22,119 seismic events using the two-band template matching scheme. The detected events have a typical duration of around 50 s and an average peak vertical velocity of approximately $1 \cdot 10^{-5}$ m/s. Event waveforms vary in shape, indicating varied sources and propagation paths. Many of the events exhibit characteristic dispersion between 0.05 and 1 Hz with high frequencies arriving before low frequencies, while others were monochromatic between 0.05 and 1 Hz.

2.2. Waveform Clustering

Because the catalog of detected events contains both dispersive and monochromatic waveforms, we seek to cluster the events into groups based on wave shape. To do so, we modify the K-shape algorithm of Paparrizos and Gravano (2016). K-shape is designed specifically to cluster time series data. Instead of calculating the Euclidean distance between potential cluster centers and observations, K-shape calculates distances using the maximum normalized cross correlation coefficient between two time series. We adapt the K-shape algorithm for three component seismic data by independently computing the cross-correlation time series between the three separate seismic channels (vertical, East, and North). We then sum these three cross-correlation time series and calculate the distance metric as the maximum value of this summed cross correlation time series.

We use the K-shape algorithm to divide the catalog into $2, 3, \cdots, 20$ clusters. However, beyond two clusters, the differences between waveforms in each cluster become progressively less clear, and an analysis of the average distance from waveforms to their cluster center does not show significant improvement for larger numbers of clusters. We thus use the K-shape algorithm to divide the catalog into two distinct clusters, which differ based on waveform dispersion. The first cluster contains 8,184 dispersive events. The second cluster contains 13,935 monochromatic events that do not exhibit dispersion within the chosen frequency band. This difference suggests that the two types of waveforms may have been generated by different source processes. Since we are specifically interested in dispersive flexural gravity wave signals, we restrict the remaining analysis to the dispersive cluster.

3. Text S3. Methods for computing event back-azimuths.

3.1. Robust first arrival determination

We obtain the relative first arrival time of each event through phase lags measurements. We cross-correlate each respective component waveform between each seismic station. We choose a window length of 500 s around the first arrival. The trace that requires the largest shift forward in time to align with the other traces is taken to be the station of first arrival. In most cases, the first arrivals obtained independently using each component are in agreement for at least two components out of three. However, if all three components produce different stations of first arrival, a back-azimuth is not calculated and the event is disregarded.

3.2. Amplitude threshold

Next, we ensure that the polarization is extracted over a high signal to noise ratio event as against noise. We slide through the event waveform in 10 s windows with a step size of five seconds. For each 10 s time window, we check if the average amplitude of that window exceeds the average amplitude of the entire 500 s event window.

3.3. Principal component analysis

For time windows with sufficiently large amplitude, principal component analysis (PCA) is performed on the HHE (East) and HHN (North) traces from each station to retrieve the PCA components. The PCA first component is a vector whose direction explains the largest contribution of the data variance. It is equivalent to the eigenvector of the data covariance matrix that has the largest eigenvalue.

3.4. PCA first component vector correction

For waves polarized in the direction of propagation, the PCA first component vector corresponds to one of the two possible propagation directions separated by 180 degrees. Using the PCA first component vector and the geometry of the array, we compute the predicted stations of first arrival corresponding to both possible propagation directions. If the station of first arrival is in the direction of the PCA back-azimuth, the PCA first component's sign is preserved. If the station of first arrival is in the opposite direction (PCA azimuth+180 degree), we add 180 degrees to the PCA first component azimuth. This ensures that the PCA first component vector points in the direction from which incoming waves arrived.

3.5. Determining the predicted first arrival

We try three methods of computing the predicted station of first arrival corresponding to both possible propagation directions.

In the first method, we compare both possible phase back-azimuths to the back-azimuths of each station with respect to the mean station location, or array centroid. The stations that are radially closest to each possible back-azimuth are predicted to be the two possible first arrivals. The sign of the PCA first component vector is then adjusted to match the propagation direction whose predicted first arrival agree with the observed first arrival. Phases for which neither predicted first arrival agreed with the observed first arrival are discarded.

In the second method, we divide the array into two sectors along a line through the array centroid orthogonal to the PCA first component vector. The sign of the PCA first

component vector is then adjusted to match the propagation direction corresponding to the sector containing the observed first arrival. No phases are discarded.

In the third method, we compute the distance vector from the array centroid to each station. For incoming plane waves, the station farthest from the array centroid in the direction of propagation records the first arrival. The stations whose distance vectors have the largest component oriented in each possible propagation directions are predicted to be the two possible first arrivals. The sign of the PCA first component vector is then adjusted to match the propagation direction whose predicted first arrival agree with the observed first arrival. Phases for which neither predicted first arrival agreed with the observed first arrival are discarded. All three methods gave relatively consistent results.

3.6. Back-azimuth stacking

Next, we sum the PCA first component vectors across each station to obtain an average vector whose norm indicates the level of agreement between propagation directions calculated at each station. Finally, we take the arctangent of the quotient of the two elements of the PCA component vector to retrieve a back-azimuth. Because this procedure is repeated for each 10 s time window in the event, the result for each individual event is a distribution of back-azimuths calculated for each time window within that event.

To obtain a single back-azimuth for each event, we take the average of the back-azimuths calculated using each time window in the data. We use the mean of circular quantities, with the back-azimuth from each time window weighted by the norm of the summed PCA components across the array for that window. This means that time windows with poor agreement between stations are downweighted when taking the average back-azimuth. The

weighted mean of circular quantities is expressed below for the back-azimuth distribution $\theta_1, ..., \theta_n$ with PCA norms $w_1, ..., w_n$ of a single event with n time windows:

$$\bar{\theta} = \text{atan2}\left(\frac{1}{n}\sum_{j=1}^{n} w_j \sin(\theta_j), \frac{1}{n}\sum_{j=1}^{n} w_j \cos(\theta_j)\right) \tag{1}$$

3.7. Uncertainties in icequake back-azimuths

Uncertainties for icequake back-azimuths were computed using the weighted standard deviation of circular quantities for each event. The weighted standard deviation of circular quantities is expressed below for the back-azimuth distribution $\theta_1, ..., \theta_n$ with PCA norms $w_1, ..., w_n$ of a single event with n time windows:

$$\sigma = \sqrt{\left(\frac{1}{n}\sum_{j=1}^{n} w_j \sin(\theta_j)\right)^2 + \left(\frac{1}{n}\sum_{j=1}^{n} w_j \cos(\theta_j)\right)^2} \tag{2}$$

The mean back-azimuthal uncertainty for rift-tip icequakes was 26.16°. The mean back-azimuthal uncertainty for rift/margin icequakes was 17.26°. The mean back-azimuthal uncertainty for shear margin icequakes was 20.13°.

The slight mislocation of the rift-tip icequake back-azimuths apparent in Figure 2 is explained in a few ways. First, imagery suggests that much of the rift-tip fracture in 2013 occurred at the large wing extending south of the rift axis. The distribution of rift-tip seismic event back-azimuths points just south of this crack by $\sim 1°$. We note that the LANDSAT image displayed in Figure 2 is from October 12, 2013, about a month before the large swarm of rift-tip seismicity in November 2013. Because the wing crack continued to advance and widen in the month after the LANDSAT image was captured, we expect fracturing and seismicity to have occurred in front of the wing crack tip's

position in Figure 2. Second, the correspondence in time between the large swarm of rift-tip icequakes in November 2013 and visible fracture evolution at the rift-tip wing crack suggests that the recorded icequakes were generated by that fracture. Third, seismicity in front of a rift's visible extent was observed at the Amery Ice Shelf by Bassis et al. (2007) and may indicate that the wing crack was more advanced at depth than at the surface, explaining events whose back-azimuths point just in front of the crack. Finally, no other areas consistent with distribution of rift-tip icequake back-azimuths contain any visible fracture opening or growth, suggesting that the large amount of fracture evolution at the rift tip observed in imagery is the most likely source of the recorded icequakes.

4. Text S4, Flexural gravity wave model

4.1. Analytical Solution for Ocean Surface Waves

We examine the water velocity potential function ϕ and relate it to the vertical ice shelf velocity w. We first solve the ocean surface wave equation for a body of water with infinite length and finite depth:

$$\frac{\partial^2 \phi}{\partial x^2} + \frac{\partial^2 \phi}{\partial y^2} = 0 \tag{3}$$

over the interval $-\infty < x < \infty, -h_w < y < 0$. We enforce zero velocity at the ocean floor and couple vertical velocity to the rate of beam deflection at the ocean surface:

$$\frac{\partial \phi}{\partial y}\Big|_{y=-h_w} = 0 \qquad\qquad \frac{\partial \phi}{\partial y}\Big|_{y=0} = \frac{\partial w}{\partial t} \tag{4}$$

We enforce the Sommerfeld radiation condition:

$$\begin{aligned} \phi\big|_{x\to-\infty} &= \frac{\partial \phi}{\partial x}\Big|_{x\to-\infty} = 0 \\ \phi\big|_{x\to\infty} &= \frac{\partial \phi}{\partial x}\Big|_{x\to\infty} = 0 \end{aligned} \tag{5}$$

We employ the Fourier Transform, written for an arbitrary function $f(x)$ as

$$\bar{f}(k) = \int_{-\infty}^{\infty} f(x)e^{-i\xi x}dx$$

Applying the Fourier Transform in x to each term of Equation 3 yields:

$$\int_{-\infty}^{\infty} \frac{\partial^2 \phi}{\partial x^2} e^{-i\xi x}dx + \frac{\partial^2 \bar{\phi}}{\partial y^2} = 0$$

Evaluating the integral term:

$$e^{-i\xi x}\frac{\partial \phi}{\partial x}\Big|_{-\infty}^{\infty} + i\xi e^{-i\xi x}\phi\Big|_{-\infty}^{\infty} - \xi^2\bar{\phi} \ + \frac{\partial^2 \bar{\phi}}{\partial y^2} = 0$$

We then apply the radiation condition (Equation 5) to eliminate the first two terms, resulting in an ordinary differential equation of $\bar{\phi}$:

$$-\xi^2\bar{\phi} \ + \frac{\partial^2 \bar{\phi}}{\partial y^2} = 0 \tag{6}$$

By applying the vertical boundary conditions (Equation 4), we obtain the time-wavenumber domain solution that satisfies the governing equation and boundary conditions:

$$\bar{\phi} = \frac{\partial \bar{w}}{\partial t}\left(\frac{\cosh(\xi(h_w + y))}{\xi \sinh(h_w \xi)}\right) \tag{7}$$

We note that ϕ is a linear function of w, therefore permitting us to write the floating beam equation using the linear operator $\mathcal{A}$ as noted in the main text.

4.2. Analytic Solution for Buoyant Ice Shelf Flexure

To interrogate the source process that explains the observations, we obtain the Green's function, or fundamental solution of a floating dynamic beam to an impulse forcing. We

obtained the Green's function by using integral transform methods to solve the governing equation for an impulse forcing in space and time. We write the Green's function formulation of Equation 1 from the main text:

$$\rho_i h_i \frac{\partial^2 G}{\partial t^2} + D\frac{\partial^4 G}{\partial x^4} + \rho_w g G + \rho_w \frac{\partial \phi}{\partial t} = \delta(x)\delta(t) \tag{8}$$

where G is the Green's function, $\delta(x)$ is Dirac delta function in space, and $\delta(t)$ is the Dirac delta function in time. As before, we apply the Fourier Transform in space to each term. Next, we apply the Laplace transform, defined as,

$$g^*(s) = \int_0^\infty g(t)e^{-st}dt$$

Applying both the Fourier and Laplace Transforms to Equation 8 yields:

$$\rho_i h_i s^2 \bar{G}^* + D\xi^4 \bar{G}^* + \rho_w g \bar{G}^* + \rho_w \gamma s^2 \bar{G}^* = 1$$

We can then solve for $\bar{G}^*$ algebraically:

$$\bar{G}^* = \frac{\frac{1}{\rho_i h_i + \rho_w \gamma}}{\frac{D\xi^4 + \rho_w g}{\rho_i h_i + \rho_w \gamma} + s^2} \tag{9}$$

Finally, we analytically compute the inverse Laplace transform of Equation 9 to obtain the Fourier-transformed Green's function,

$$\bar{G}(k,t) = \frac{\sin\left(t\sqrt{\frac{D\xi^4 + \rho_w g}{\rho_i h_i + \rho_w \gamma}}\right)}{\sqrt{\rho_i h_i + \rho_w \gamma}\sqrt{D\xi^4 + \rho_w g}} \tag{10}$$

In practice, we numerically calculate $\bar{G}$ for a range of times and wavenumbers that define the temporal and spatial domain of the model run. Once $\bar{G}$ is calculated for each element of a vector of times and a vector of wavenumbers, the IFFT (inverse fast Fourier

transform) is taken to numerically retrieve the Green's function $G(x,t)$ of the ice shelf for an applied unit point force.

4.3. Greens function for a point moment source

To retrieve the impulse response to a point bending moment source, we note that an applied bending moment is equivalent to a pair of infinitesimally-spaced point loads with opposite signs:

$$G_m(x,t) = [G(x,t) - G(x+\Delta x,t)]_{\Delta x \to 0}$$
$$G(x,t) = \Delta x \left[\frac{G(x,t) - G(x+\Delta x,t)}{\Delta x} \right]_{\Delta x \to 0}$$
$$G(x,t) = \frac{dG(x,t)}{dx}$$

To obtain $G_m(x,t)$, we numerically take the spatial derivative of the point load Green's function $G(x,t)$.

4.4. Deconvolution procedure

We calculate source load through the deconvolution,

$$P_{\text{estimated}}(t) = \mathcal{F}^{-1} \left[\frac{\hat{w}(\omega)_{\text{observed}}}{\hat{G}(x_0,\omega)} \right], \tag{11}$$

where hats denote Fourier-transformed quantities, $\mathcal{F}^{-1}$ is the inverse Fourier transform, $w_{\text{observed}}(t)$ is a linear stack of observed displacement seismograms, $P_{\text{estimated}}(t)$ is an estimated source load distribution, and x_0 is the station epicentral distance. We obtain $w_{\text{observed}}(t)$ for each spatial group by aligning each waveform in the group with respect to a master event using cross correlation and taking the average waveform. Master events were selecting by finding the event from each spatial group that was best-correlated with

the overall centroid of the dispersive cluster. We choose x_0 corresponding to the average distance to each spatial group: for the rift tip, $x_0 = 25$ km; for rift/margin, $x_0 = 25$ km; for margin icequakes, $x_0 = 17.5$ km. We alternatively consider a bending moment source through the relationship,

$$M_{\text{estimated}}(t) = \mathcal{F}^{-1}\left[\frac{w(\omega)_{\text{observed}}}{G_m(x_0,\omega)}\right]. \tag{12}$$

5. Text S5, Estimating vertical crack extent from point load magnitude

The point load applied during vertical crack growth arises from the difference in ice overburden stress above the crack and buoyancy stress exerted by the water that fills the crack. Therefore, we write the following expression and substitute in the each stress:

$$\begin{aligned} P &= \sigma_{\text{overburden}} - \sigma_{\text{buoyancy}} \\ P &= \rho_i g(h_i - \Delta z_c) - \rho_w g(z_w - \Delta z_c) \end{aligned} \tag{13}$$

where P is the applied point load, ρ_i is the density of ice, ρ_w is the density of water, h_i is the ice shelf thickness, Δz_c is the change in vertical position of the crack tip, and z_w is the position of the water line.

Substituting the expression for the water line position $z_w = \frac{\rho_i}{\rho_w} h_i$ and simplifying gives:

$$P = g\Delta z_c(\rho_i - \rho_w) \tag{14}$$

Finally, we write:

$$\Delta z_c = \frac{P}{g(\rho_i - \rho_w)} \tag{15}$$

Table S1.

References

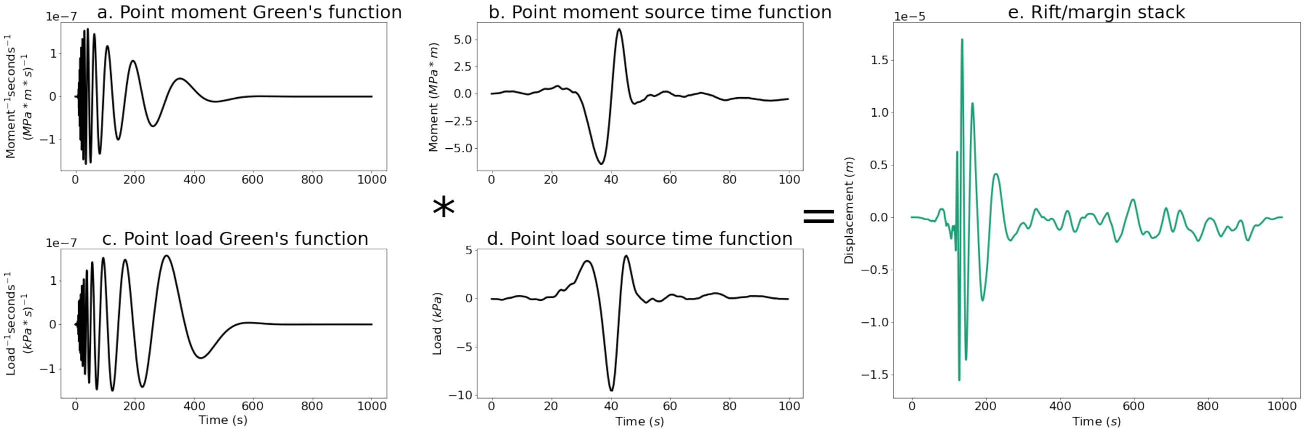

Figure S1. Green's functions and source time functions for rift/margin events. (a) Theoretical Green's function for a bending moment source located at a distance of 30 km, which is approximately the distance from PIG seismic array to the rift/margin area. (b) Source time function retrieved by deconvolving the moment Green's function from the stack of rift/margin vertical displacement waveforms. (c) Theoretical Green's function for a point load source located at a distance of 30 km, which is approximately the distance from PIG seismic array to the rift/margin area. (d) Source time function retrieved by deconvolving the point load Green's function from the stack of rift/margin vertical displacement waveforms. (e) Stack of rift/margin vertical displacement waveforms obtained by aligning waveforms to a master event and taking the mean waveform on the frequency band 0.01-1 Hz.

Table S1. Parameters for building the event catalog.

Parameter	Low Frequency Band	High Frequency Band
STA/LTA band	0.01-1 Hz	1-10 Hz
Short window (ST) length	10 s	10 s
Long window (LT) length	300 s	300 s
Trigger STA/LTA threshold	8 s	20 s
Template matching band	0.05-1 Hz	1-10 Hz
Trigger cross correlation threshold	0.3	0.2
Minimum number of stations for a detection	3	3

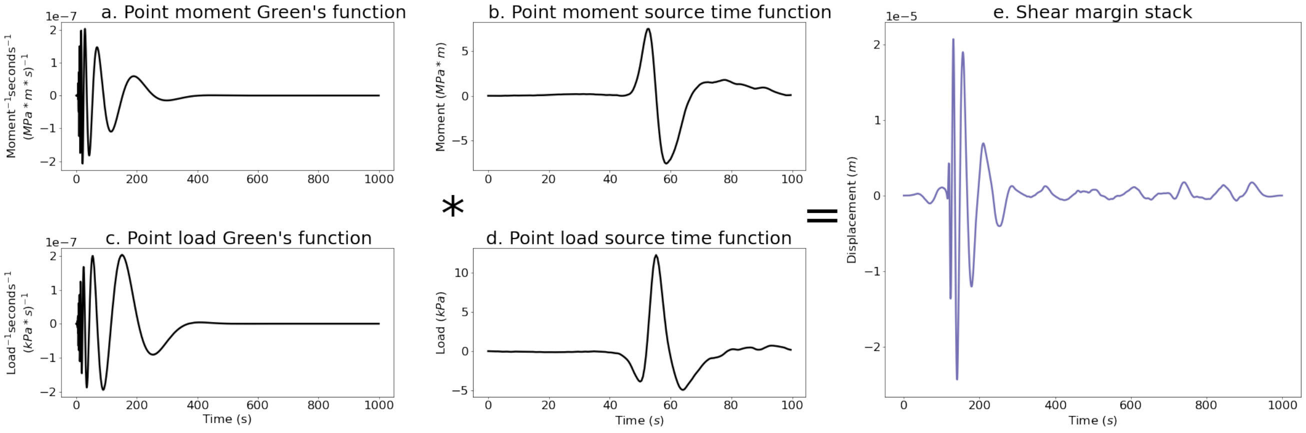

Figure S2. Green's functions and source time functions for shear margin events. (a) Theoretical Green's function for a bending moment source located at a distance of 17.5 km, which is approximately the distance from PIG seismic array to the northeast shear margin near Evans Knoll. (b) Source time function retrieved by deconvolving the moment Green's function from the stack of shear margin vertical displacement waveforms. (c) Theoretical Green's function for a point load source located at a distance of 17.5 km, which is approximately the distance from PIG seismic array to the shear margin. (d) Source time function retrieved by deconvolving the point load Green's function from the stack of shear margin vertical displacement waveforms. (e) Stack of shear margin vertical displacement waveforms obtained by aligning waveforms to a master event and taking the mean waveform on the frequency band 0.01-1 Hz.

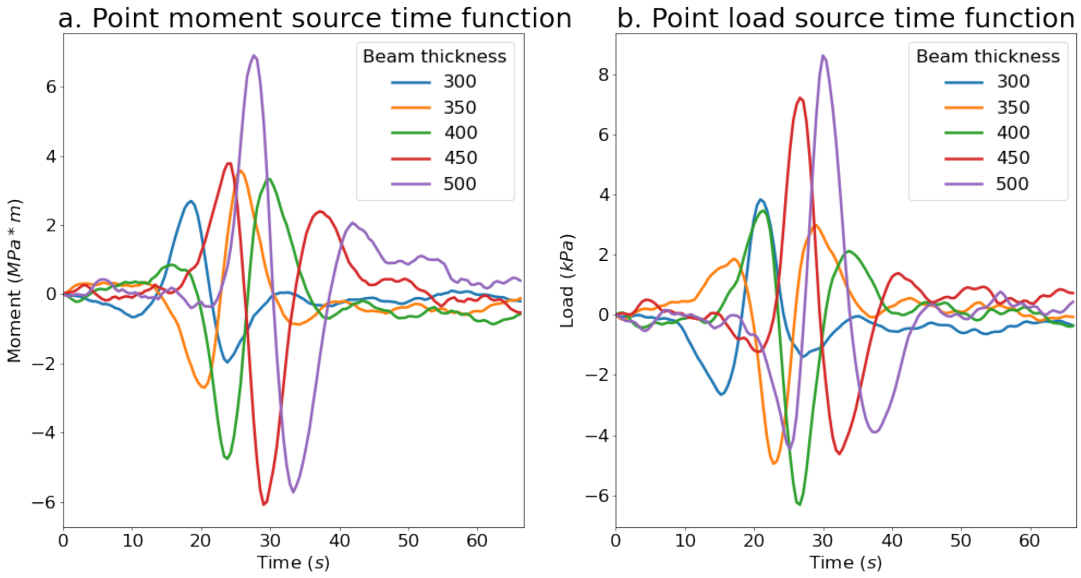

Figure S3. Sensitivity of rift tip source time function deconvolution to modeled ice thickness. Modeled beam thicknesses are shown in the legend. Source time functions generally have larger amplitude and longer duration for thicker beams, because larger forcing is required to induce a given displacement for a more rigid beam. Flexural rigidity, the parameter that governs flexure, is a function of thickness.

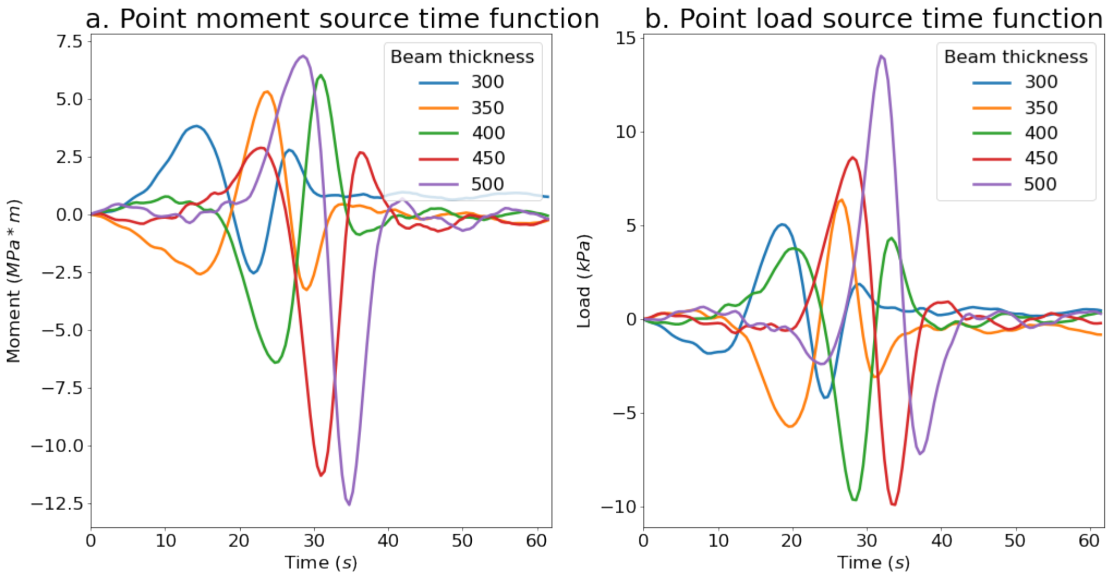

Figure S4. Sensitivity of rift/margin source time function deconvolution to modeled ice thickness. Modeled beam thicknesses are shown in the legend. Source time functions generally have larger amplitude and longer duration for thicker beams, because larger forcing is required to induce a given displacement for a more rigid beam. Flexural rigidity, the parameter that governs flexure, is a function of thickness.

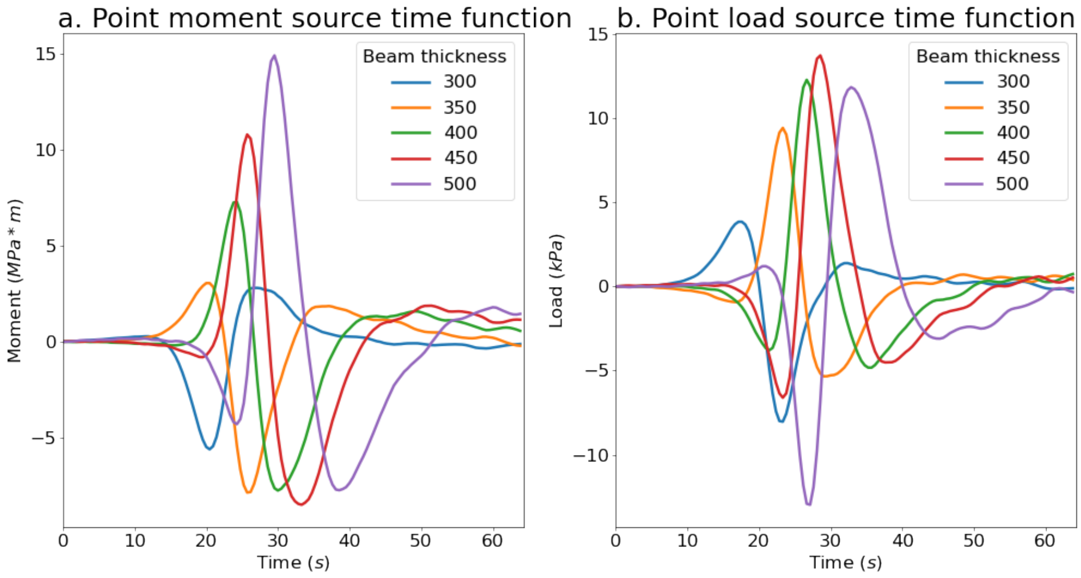

Figure S5. Sensitivity of margin source time function deconvolution to modeled ice thickness. Modeled beam thicknesses are shown in the legend. Source time functions generally have larger amplitude and longer duration for thicker beams, because larger forcing is required to induce a given displacement for a more rigid beam. Flexural rigidity, the parameter that governs flexure, is a function of thickness.

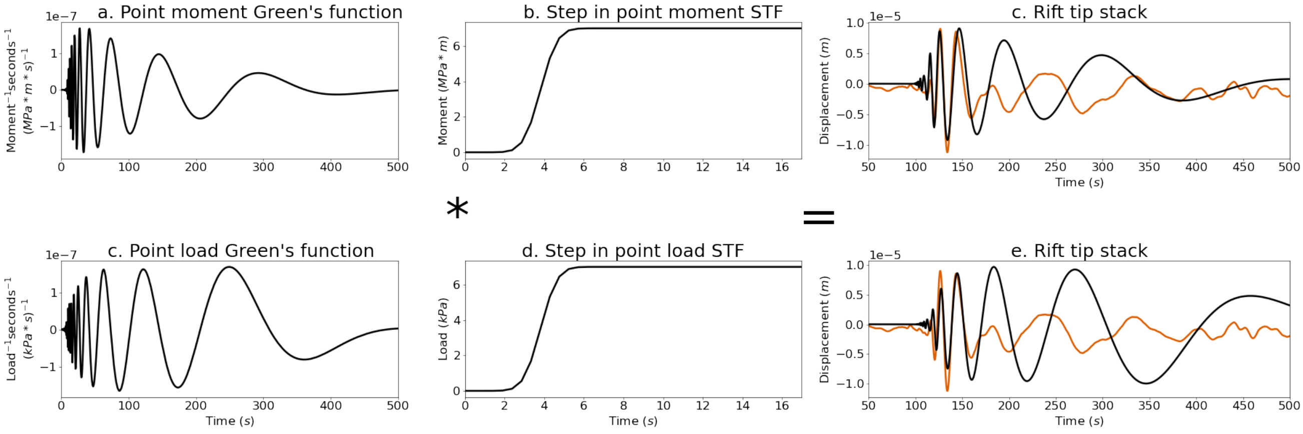

Figure S6. Modeled rift tip Green's function convolved with step source time function. The resulting modeled displacements, shown in black, have a longer decay and larger amplitdue low-frequency displacements than the rift tip stack, shown in orange, for both bending moment and point load sources.

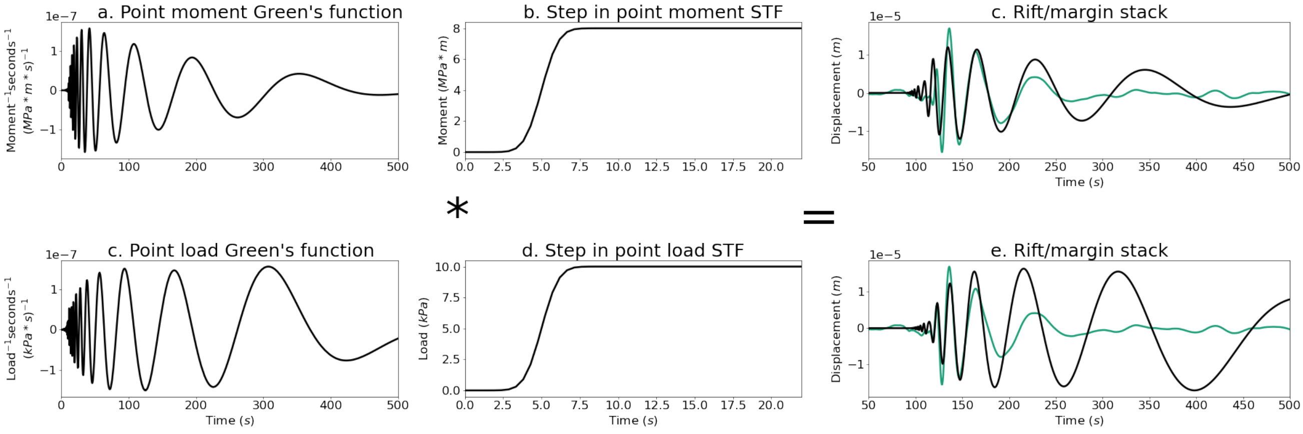

Figure S7. Modeled rift/margin Green's function convolved with step source time function. The resulting modeled displacements, shown in black, have a longer decay and larger amplitdue low-frequency displacements than the rift/margin stack, shown in green, for both bending moment and point load sources.

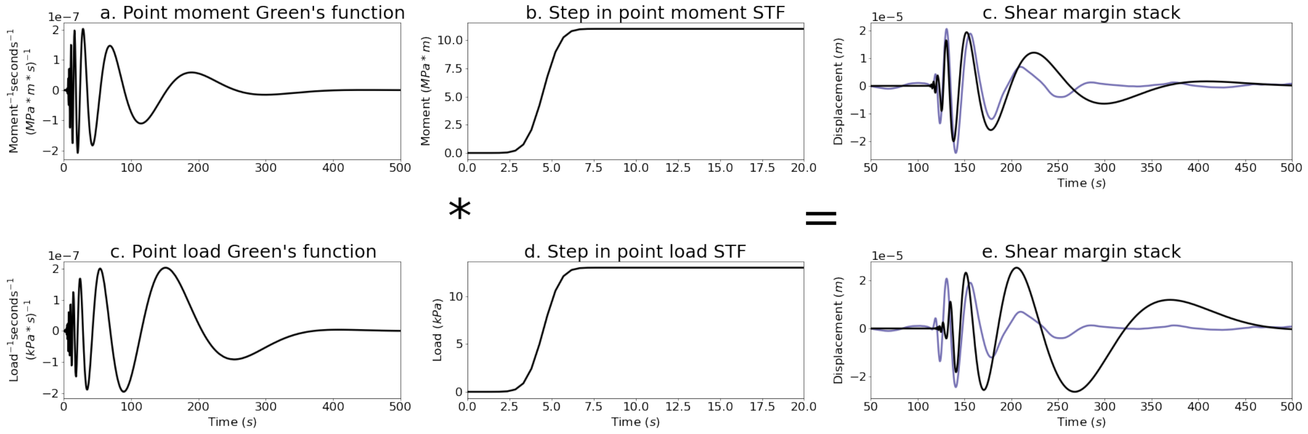

Figure S8. Modeled shear margin Green's function convolved with step source time function. The resulting modeled displacements, shown in black, have a longer decay and larger amplitdue low-frequency displacements than the shear margin stack, shown in purple, for both bending moment and point load sources. The modeled displacements arising from an applied bending moment are relatively similar to the shear margin stack, but the results of deconvolution do not support the hypothesis that the observations were generated by a step forcing in bending moment.

Allen, R. V. (1978, 10). Automatic earthquake recognition and timing from single traces. *Bulletin of the Seismological Society of America*, *68*(5), 1521-1532. Retrieved from https://doi.org/10.1785/BSSA0680051521 doi: 10.1785/BSSA0680051521

Gibbons, S. J., & Ringdal, F. (2006, 04). The detection of low magnitude seismic events using array-based waveform correlation. *Geophysical Journal International*, *165*(1), 149-166. Retrieved from https://doi.org/10.1111/j.1365-246X.2006.02865.x doi: 10.1111/j.1365-246X.2006.02865.x

Paparrizos, J., & Gravano, L. (2016, June). K-shape: Efficient and accurate clustering of time series. *SIGMOD Rec.*, *45*(1), 69–76. Retrieved from https://doi.org/10.1145/2949741.2949758 doi: 10.1145/2949741.2949758
